# Seven cases of probable endotoxin poisoning related to contaminated glutathione infusions

**DOI:** 10.1017/S0950268818000420

**Published:** 2018-04-20

**Authors:** T. Johnstone, E. Quinn, S. Tobin, R. Davis, Z. Najjar, B. Battye, L. Gupta

**Affiliations:** 1 Public Health Unit, Population Health Unit, Sydney Local Health District, Sydney, Australia; 2 Health Protection NSW, NSW Ministry of Health, Sydney, Australia; 3 Royal Prince Alfred Hospital, Sydney Local Health District, Sydney, Australia; 4 Pharmaceutical Regulatory Unit, NSW Ministry of Health, Sydney, Australia

**Keywords:** cluster, compounded pharmaceutical, Endotoxin, glutathione

## Abstract

We report seven cases of probable endotoxin poisoning linked to contaminated compounded glutathione. Five of the cases were using the infusions for treatment of Lyme disease highlighting the risks of using compounded sterile preparations for unapproved indications, especially if the quality of source products cannot be assured.

## Background

Compounded sterile preparations (CSPs) are therapeutic products made by pharmacists within the community or hospital pharmacies. Sterility is highly dependent on the production process; hence there is a subsequent risk to patients of contamination from poor quality control in the production process of either the source product or the compounded preparation itself [1, 2]. In the last decade, cases and outbreaks of bloodstream infections, bacterial sepsis, endotoxin-related systemic inflammatory response syndrome and other infections have been reported predominantly in the USA, related to contaminated CSPs [1–5]. This is the first report of a cluster of adverse events related to endotoxin-contaminated glutathione preparations. We describe the public health investigation, including the trace-back of the CSPs and actions taken to avert further adverse events when contamination of this product was suspected.

In February 2015, a local public health authority in Sydney, in the state of New South Wales (NSW), was notified of a 41-year-old female patient who presented to an emergency department with symptoms consistent with bacterial sepsis. She had received a glutathione and phosphatidylcholine infusion at a nearby complementary medicine clinic that day and reported feeling ill 15 min into infusion administration with symptoms of fever (39.5 °C), hypotension (BP 79/47), muscle twitches, vomiting, diarrhoea and abdominal, neck and back pain. The hypotension persisted despite administration of 4 l normal saline. The patient had been receiving these infusions weekly, prescribed by her general practitioner (GP), who had diagnosed her with Lyme disease and *Bartonella* and *Babesia* infection. After presenting to the emergency department, the patient was treated with broad-spectrum antimicrobial therapy for possible bacterial septicaemia, with a differential diagnosis of endotoxin-related systemic inflammatory response syndrome. Although blood and urine cultures were negative, the severity of the clinical picture and difficulty in excluding bacterial sepsis led to antibiotic treatment for 8 and 5 days of hospitalisation. At a follow-up appointment on day 15 clinical improvement was noted. However, she developed *Clostridium difficile* associated diarrhoea on day 17, with *C. difficile tcdB* gene detected in stool, a likely complication of hospitalisation and administration of broad-spectrum antibiotics.

During admission, the patient reported that other patients at the complementary medicine clinic had experienced similar reactions. The hospital staff notified this to the local public health authority, which then initiated an investigation after confirmation from the clinic nurse that five patients had recently experienced similar symptoms after a glutathione infusion acquired from the same pharmacy.

On the day after notification of the index case, an investigation team consisting of NSW Ministry of Health staff and two local public health authorities was established. The objectives of the investigation were to identify if there was a cluster of illness related to this product, identify a source, and rapidly prevent any further cases if a source was identified.

## Methods

Active surveillance for cases was initiated across NSW hospital microbiology departments whereby laboratory staff were asked to report any cases of suspected systemic inflammatory response syndrome or sepsis without a known bacterial cause to the relevant public health authority. A ‘case’ in this investigation was defined as a person who received glutathione parenteral products made from the same batch of source product as the index case and subsequently developed symptoms within 24 h of receiving the infusion. Cases were interviewed using a questionnaire developed for the purpose of administering by telephone. The questionnaire collected demographic and epidemiological data, including symptom profile, time of symptom onset and duration of illness.

On day 2 of the investigation, staff at the pharmacy from which the cases had obtained their infusions were interviewed about their compounding procedures and samples of the source glutathione powder used to prepare the CSP were collected. A list of all patients who had been dispensed glutathione products in the 6 weeks prior to the onset of the index case was obtained. The complementary medicine clinic was inspected on day 2 to assess the infusion administration technique and infection control practices. An independent pharmaceutical consultant audited the pharmacy's manufacturing processes for compounding on day 15.

A 200 g sample of the glutathione powder from the batch used to compound the preparations for all cases was collected and sent to the national pharmaceutical regulator, the Therapeutic Goods Administration (TGA) and the state Forensic and Analytical Science Service for chemical and microbiological testing. Seven vials of glutathione which had been dispensed from the pharmacy, to be infused at the clinic, but not yet administered, were collected from patients. Six of these vials were sent to the TGA for microbiological and endotoxin testing and one, from the index case, remained at the hospital for culturing. Toxin testing of clinical samples was unavailable.

## Results

The pharmacy had made 68 compounded glutathione products (33 creams, 27 parenteral preparations and eight inhalants) in the 6 weeks prior to the onset of the index case, which were dispensed to 52 patients. Patients were prescribed a glutathione parenteral product from their GP; the product was then compounded as prescribed and dispensed by the pharmacy, stored by the patient at their home and then administered at the complementary medicine clinic, with a range of 1–13 days between dispensing and administration of the product (Table 1). This timeframe is particularly relevant given that glutathione for injection has an expiry date of 21 days.

**Table 1.** Profile of the preparation, administration and symptom onset of the seven patients receiving compounded glutathione infusions prepared by a pharmacy in Sydney, Australia, February 2015

Case number	Dispensing day^a^	Administration day^a^	Glutathione	Phosphati dylcholine	Vitamin C	Onset	Fevers	Rigors	Head aches	Nausea	Sweating	Vomiting	Diarrhoea	Abdominal pain	Chest pain	Hypo tension	Light headed	Loss of consciousness	Duration of symptoms (days)	Presented to emergency department	Presented to GP
1	−7	1	X	X		15 min into infusion	X	X	X			X	X	X					5	X	
2	−6	−5	X		X	10 min after infusion	X	X	X	X	X	X				X	X		4		X
3	−12	−5	X	X	X	30 min after infusion	X	X	X	X	X	X	X	X	X				4		
4	−13	1	X		X	30 min after infusion	X	X			X		X		X				3	X	
5	−12	1	X	X	X	2 h after infusion	X	X	X		X								2		
6	−7	−5	X		X	1 h after infusion	X	X	X	X		X							3		
7	−10	−5	X		X	30 min into infusion	X	X	X	X						X	X	X	7		

a Dispensing and administration day are reported in relation to start of investigation (day 1).

Of these 52 patients, nine had been prescribed glutathione parenteral products made from the same batch of glutathione as the index case. Seven of these nine patients met the investigation case definition. The remaining two were dispensed the product, but had not been administered the preparation; one because of pain at the cannula site prior to the commencement of the infusion and one who had been contacted by the pharmacy and notified of the problem prior to infusion.

Of the seven cases, six received the infusions at the same complementary medicine clinic and one received it from their local doctor intrastate. All patients received glutathione with vitamin C and/or phosphatidylcholine in their infusions and were symptomatic within 2 h of administration with an average duration of illness of 3 days (Table 1). Most cases reported fever, rigours and headaches (Table 1). Five of the seven patients were prescribed glutathione from their GP for treatment of Lyme disease.

During the first pharmacy inspection, it was noted that staff had received complaints of adverse reactions from the complementary medicine clinic nurse. Based on this information, the pharmacy had recalled all glutathione products made using the same batch as the index case, which was purchased from a new overseas manufacturer. No concerns regarding infection control were identified in the inspection of the complementary medicine clinic, and the nurse agreed to not administer any further glutathione infusions until the source of the problem was identified. The pharmacy audit conducted by the independent consultant identified a range of issues with the aseptic production of compounded products, including minimal compliance with national quality assurance guidelines.

Testing of the glutathione powder and infusions revealed no chemistry quality issues or microbiological contamination. However, endotoxin testing of the seven unused vials and powder sample revealed high levels of endotoxins in all samples, exceeding the internationally accepted maximum pyrogenic threshold of 5 Endotoxin units/kg/h (Ph. Eur. 5.1.10) [6] and therefore failing the requirements for Parenteral Preparations in the European and British Pharmacopoeia and Therapeutic Goods Order 77 [7]. Testing of vials from an alternate batch of glutathione powder from a different supplier revealed no endotoxin contamination.

## Discussion

Our investigation demonstrated convincing toxicological and epidemiological evidence that this cluster of adverse reactions was caused by endotoxin-contaminated CSPs administered as a parenteral infusion. A diagnosis of endotoxin-induced systemic inflammatory response syndrome was made on the index case based on clinical presentation, the absence of a causative organism for bacterial sepsis and the history of a recent infusion. The identification of the cluster led to the removal of the suspected batch of the product from circulation and potentially avoided further cases of endotoxin-mediated illness in patients. Despite this rapid investigation, it is important to note that earlier notification of these adverse reactions to the local public health authority from the pharmacy or clinic may have averted at least four of the cases in this investigation, as they had received their infusions and experienced symptoms 6 days prior to the index case presentation (Table 1).

This investigation exposed potential issues with the global manufacturer of active pharmaceutical ingredients and their importation for subsequent use in compounded products. Although we could not identify the location and time of the contamination or the causative pathogen, it is very likely that some gram-negative bacteria produced the endotoxin while growing in the solution used to chemically synthesise glutathione by the overseas manufacturer. In addition, an exemption in the Australian *Therapeutic Goods Act 1989* has meant that there is no requirement for CSPs, such as glutathione infusions, to be regulated by the TGA [8]. Since 2013, the TGA has been considering various options for the regulatory reform of compounding pharmacies, including that compounded parenteral pharmaceuticals, are only produced by TGA-licensed manufacturers and have a short expiry date.

In addition, this cluster highlights the risks of prescribing medications for administration via routes that have not been approved and when there is insufficient high-quality evidence for their use. In Australia, glutathione is listed by the TGA as a safe active ingredient for use in adults as an oral dose [9], but not as a parenteral preparation. Further, robust high-quality evidence to demonstrate the efficacy of glutathione for immune-compromised persons [10] or those diagnosed with Lyme borreliosis [11] does not exist. Prescribers should consider the evidence with regard to the use of CSPs from community pharmacies, and weigh the potential risks with the benefits when using these products, especially if regulation of preparation and quality control of CSPs from community pharmacies is limited.

## Summary

We report seven cases of probable endotoxin poisoning across New South Wales, Australia following infusions of compounded glutathione. The public health investigation identified the source of the cluster as contaminated glutathione powder used in the production of the infusions which were produced by a single compounding pharmacy in Sydney, Australia using imported active ingredients. The contamination likely occurred prior to the glutathione powder being delivered to the pharmacy underscoring the importance of rigorous quality assurance practices when importing pharmaceutical ingredients. Five of the cases were using the infusions for treatment of Lyme disease highlighting the risks of parenteral administration of compounded sterile preparations for unapproved indications, especially if the quality of source products cannot be assured. This paper adds to the literature describing the need for pharmaceutical regulation to keep pace with the growing use of compounded pharmaceutical products and the importance for physicians to understand the potential infection risks associated with prescribing compounded pharmaceutical products.
